# Prohibitin: a potential therapeutic target in tyrosine kinase signaling

**DOI:** 10.1038/sigtrans.2017.59

**Published:** 2017-12-15

**Authors:** Sudharsana Rao Ande, Yang Xin Zi Xu, Suresh Mishra

**Affiliations:** 1 Department of Internal Medicine, University of Manitoba, Winnipeg, Manitoba, Canada; 2 Department of Physiology & Pathophysiology, University of Manitoba, Winnipeg, Manitoba, Canada

## Abstract

Prohibitin is a pleiotropic protein that has roles in fundamental cellular processes, such as cellular proliferation and mitochondrial housekeeping, and in cell- or tissue-specific functions, such as adipogenesis and immune cell functions. The different functions of prohibitin are mediated by its cell compartment-specific attributes, which include acting as an adaptor molecule in membrane signaling, a scaffolding protein in mitochondria, and a transcriptional co-regulator in the nucleus. However, the precise relationship between its distinct cellular localization and diverse functions remain largely unknown. Accumulating evidence suggests that the phosphorylation of prohibitin plays a role in a number of cell signaling pathways and in intracellular trafficking. Herein, we discuss the known and potential importance of the site-specific phosphorylation of prohibitin in regulating these features. We will discuss this in the context of new evidence from tissue-specific transgenic mouse models of prohibitin, including a mutant prohibitin lacking a crucial tyrosine phosphorylation site. We conclude with the opinion that prohibitin can be used as a potential target for tyrosine kinase signal transduction-targeting therapy, including in insulin, growth factors, and immune signaling pathways.

## Introduction

Prohibitin (PHB or PHB1) was first identified as an anti-proliferative gene and its cDNA was isolated by differential hybridization to RNA from normal versus regenerating liver tissue in rats.^1^ The *PHB1* gene is present on the chromosome 17q21 locus, a region that is associated with a propensity for visceral fat deposition in humans.^2^ Microinjection of PHB1 mRNA into normal human fibroblasts attenuated DNA synthesis and led to their growth inhibition.^3^ Later, it was discovered that growth inhibition caused by PHB1 mRNA was due to the 3′ untranslated region and not the coding region.^4^ The PHB1 protein is a member of a highly conserved family of proteins known as the Band-7 or prohibitin domain family, which also includes the repressor of estrogen activity (REA or PHB2), stomatins, HflK/C, flotillins, and a plant defense protein family known as HIR (hypersensitive induced reaction).^5^ There are three major domains in the protein sequence of PHB1: the N-terminal hydrophobic alpha helix that functions as a membrane-anchoring domain, the mid-region that contains the PHB domain, and the C-terminus that contains a coiled-coil domain with a small nuclear localization sequence.^6^ PHB1 has been implicated in diverse fundamental cellular functions, such as cell proliferation,^7^ cell cycle control,^8^ differentiation^9^ and protection from oxidative stress,^10^ and it acts as a molecular chaperone.^11^ PHB1 is a ubiquitously expressed protein that is present in various cellular compartments, such as the cell membrane,^12^ mitochondria^7,13^ and nucleus.^14^ Although PHB1 was discovered >25 years ago, little is known about the regulation of this protein. Unfortunately, global knockdown of PHB1 leads to embryonic lethality in multicellular organisms, such as *Caenorhabditis elegans *and *Mus musculus*, which has prevented researchers from identifying and illustrating the molecular functions of PHB1 at an organismal level.^15,16^ PHB1 is known to undergo various posttranslational modifications such as *O*-GlcNAc modification,^17^ palmitoylation,^18^ ubiquitination,^19,20^ phosphorylation^13,17,21–23^ and cysteine oxidation.^24^

Post-translational modification plays an important role in the regulation, stability, trafficking and cell signaling functions of proteins. Several of the commonly occurring posttranslational modifications in proteins are known to occur at the same residue in a mutually exclusive manner or regulate nearby modification sites. For example, *O*-GlcNAc modification and serine/threonine phosphorylation that occur at the same residues in certain proteins have been shown to be regulated by tyrosine phosphorylation in nearby residues, and they can influence each other.^17^ Similarly, acetylation, ubiquitination and methylation may occur at the same lysine residue in a mutually exclusive manner and add diversity to protein functions and their regulation.^25^ Phosphorylation is the most extensively studied posttranslational modification and orchestrates a variety of cellular and molecular events, such as cell proliferation, differentiation and cell death. It has been estimated that more than 30% of cellular proteins contain covalently attached phosphate groups at a ratio of 1800:200:1 for serine, threonine and tyrosine residues, respectively.^26^ Phosphorylation is very important for normal cellular functions, while dysregulated phosphorylation has been implicated in various disease conditions such as cancer, diabetes and neurological disorders. Phosphorylation is a fundamental posttranslational modification of PHB1 that regulates its function, intracellular trafficking and its binding specificity for certain proteins. There are two major forms of phosphorylation known to occur in PHB1, serine/threonine phosphorylation and tyrosine phosphorylation. In this review, we highlight the roles of tyrosine phosphorylation of PHB1. In the entire human PHB1 protein there are only four tyrosine residues, at positions 28, 114, 249 and 259, and they are all highly conserved among different species.^27^ PHB1 is known to undergo tyrosine phosphorylation at multiple residues. There are several reports in the literature that show that PHB1 undergoes tyrosine phosphorylation upon activation by various stimuli.^17,21,22^ These stimuli include insulin, epidermal growth factor (EGF), platelet-derived growth factor (PDGF) and insulin-like growth factor (IGF).^21–23,28^ Recent discoveries have highlighted a crucial role for PHB1 in the MAPK/ERK pathway, PI3 kinase/Akt pathway, TGF-β signaling pathway and STAT3 signaling pathway.^21,29–31^ Herein, we describe the active role played by tyrosine phosphorylation of PHB1 in various signaling pathways and its mechanism of action in regulating these signaling molecules.

## Tyrosine phosphorylation of PHB1 and insulin signaling

Activation of the insulin signaling pathway is initiated by with insulin binding to its receptor resulting in its activation. Upon activation the insulin receptor undergoes dimerization and auto-phosphorylation of its cytoplasmic domains.^32^ These events lead to increased tyrosine kinase activity of the insulin receptor towards its substrates. Upon activation, insulin receptor phosphorylates insulin receptor substrates (IRS) and Src homologous/collagen proteins (Shc). These dynamic events lead to the activation of subsequent signaling pathways; the PI3/Akt kinase pathway and MAPK/ERK pathway.^32^ PI3 kinase consists of two subunits: a catalytic subunit, p110, and a regulatory subunit, p85.^33^ PI3 kinase promotes the conversion of phosphatidylinositol 4,5-bis phosphate (PIP2) to phosphatidylinositol 3,4,5-triphosphate (PIP3). PIP3 acts as a second messenger and activates downstream target alpha serine/threonine protein kinase (Akt). Activation of Akt leads to various biological effects including the suppression of apoptosis by inactivating pro-apoptotic molecules such as BAD, BAX and caspase-9.^34^ The other arm of the tyrosine kinase signaling pathway is activated by binding of tyrosine-phosphorylated IRS or Shc to growth factor receptor bound protein −2 (Grb-2) and Sons of sevenless (Sos).^32^ These events lead to the activation of Ras and Raf-1 signaling which eventually activates MAPK and ERK signaling and the translocation of ERK to the nucleus.^35^ Upon translocation to the nucleus, ERK activate several mitogenic factors (for example, Myc and Elk1) that lead to the survival and proliferation of cells.

**Figure 1:**
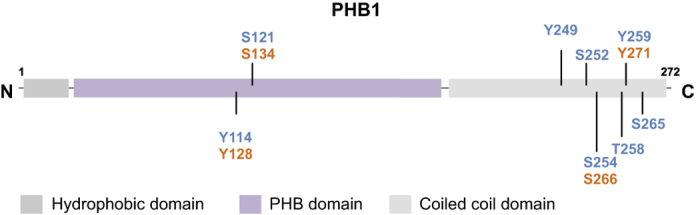
A schematic diagram showing the location of known phosphorylation sites in different structural domains in PHB1. The conserved phosphorylation sites in PHB2 are shown in red. S, serine; T, threonine; Y, tyrosine.

**Figure 2:**
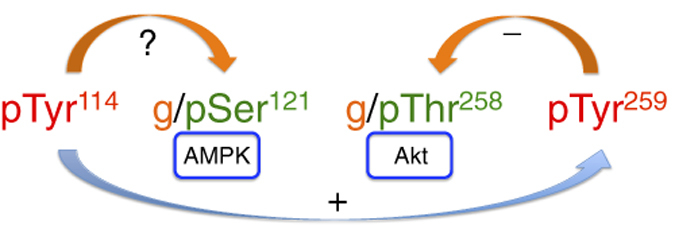
A schematic diagram showing known and potential crosstalk between tyrosine phosphorylation and serine/threonine phosphorylation or *O*-GlcNAc modification in PHB1. The phosphorylation of PHB at Tyr-114 is known to have a priming effect on the phosphorylation at Tyr-259 and Tyr-249 residues.^17^ Ser, serine; Thr, threonine; Tyr, tyrosine; g -O-GlcNAc modified; p, phosphorylated.

We have reported that the phosphorylation of PHB1 at Tyr-114 plays a role in attenuating insulin signaling.^21^ Initially, PHB1 is phosphorylated at Tyr-114 upon stimulation with insulin. Once phosphorylated, it creates a binding site for SH2 domain-containing protein tyrosine phosphatase-1 (Shp1), which consequently alters the phosphorylation of Akt and glycogen synthase kinase-3β.^21^ This finding suggests that the insulin-induced tyrosine phosphorylation of PHB1 and subsequent recruitment of Shp1 may modulate insulin receptor substrates, PI3 kinase activity and subsequent downstream insulin signaling. In addition to the insulin receptor, other tyrosine kinases that can potentially phosphorylate PHB1 at tyrosine residues include the membrane tyrosine kinases EGFR, PDGFR and IGFR as well as Src domain-containing cytoplasmic tyrosine kinases such as Lyn and Syk. In this context, it is important to note that the Tyr-114 phosphorylation site is conserved in the homologous protein PHB2 and that the corresponding residue in PHB2 is Tyr-128 (Figure 1). Thus, it is possible that the conserved phosphorylation sites in PHB1 and PHB2 can function in the same way and be regulated by a common mechanism. Furthermore, we have shown that PHB1 interacts with *O*-GlcNAc transferase (OGT) and undergoes *O*-GlcNAc modification at Ser-121 and Thr-258,^17^ which are also phosphorylation sites for AMPK and Akt, respectively (Figure 2). These two locations are in close proximity to two tyrosine phosphorylation sites, Tyr-114 and Tyr-259, in PHB1.^17^ Substitution of Tyr-114 and Tyr-259 phosphorylation sites with phenylalanine decreases *O*-GlcNAc modification in PHB1;^17^ whereas the substitution of Ser-121 and Thr-258 with alanine and isoleucine, respectively leads to increased tyrosine phosphorylation of PHB1.^17^ These findings suggest that there is a strong regulatory association between tyrosine phosphorylation and *O*-GlcNAc modification at Ser/Thr residues in PHB1 (Figure 2). Although the biological significance of *O*-GlcNAc modification of PHB1 remains unclear, its relationship with functionally relevant phosphorylation sites in PHB1 (that is, Tyr-114, Ser-121, Thr-258, and Tyr-259) indicates an important role in the regulation of PHB1 functions.^17,36^ This binary switch between tyrosine phosphorylation and *O*-GlcNAc modifications provides new mechanistic insight into various cell-signaling pathways, which warrants further investigation.

**Figure 3:**
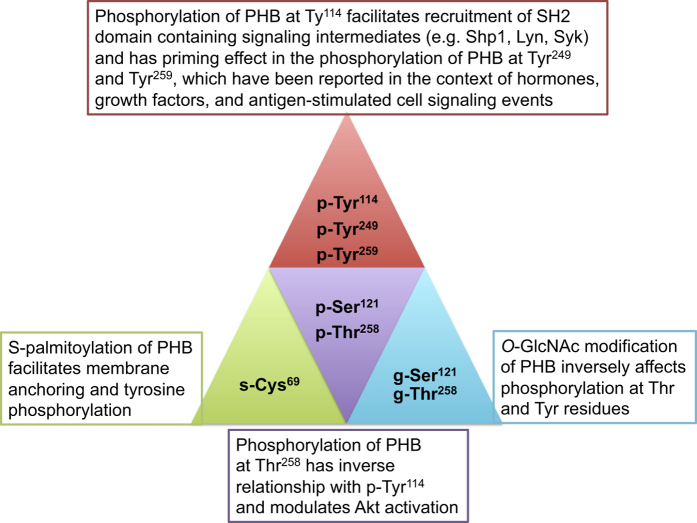
A schematic diagram showing the relationship among various posttranslational modifications of PHB1 and their relevance to the regulation of PHB1 functions and cell signaling events. Additionally, phosphorylation of PHB1 appears to be involved in the intracellular trafficking of PHB1 (not shown).

PHB1 is also known to interact with phosphatidylinositol 3,4,5-triphosphate (PIP3) to modulate insulin signaling.^22^ Analysis of the PHB1 protein sequence with BLAST for comparison with proteins with known lipid-binding domain revealed that PHB1 contains conserved lipid-binding PX domains, which may assist PHB1 in interacting with PIP3 and other lipids.^22^ The Tyr-114 phosphorylation site is in close proximity to the putative PIP3 binding domain of PHB1.^22^ Thus, it is possible that the phosphorylation status of Tyr-114 in PHB1 may influence other interacting partners of PHB1, either directly or indirectly, that have a role in PIP3 signaling. There are several reports in the literature showing PHB1 is translocated from mitochondria to the plasma membrane,^18,37^ and from the nucleus to mitochondria upon activation by various stimuli or agents.^10,38,39^ However, the mechanisms involved in the intracellular trafficking of PHB1 are not well understood. We have shown that PHB1 undergoes palmitoylation at Cys-69 and interacts with Eps 15 homology domain protein 2 (EHD2).^18^ This modification facilitates translocation of PHB1 to the membrane and its tyrosine phosphorylation.^18^ This was the first report that elucidated the mechanism behind translocation of PHB1 to the plasma membrane. Taken together, these studies suggest that the tyrosine phosphorylation of PHB1 plays an important role in modulating PI3K/Akt signaling pathways and has a relationship with neighboring post-translational modifications on the protein (Figure 3). Perturbation of tyrosine phosphorylation of PHB1 leads to impaired insulin signaling and altered cell metabolism, and these events may eventually lead to the development of type-2 diabetes and cancer. Hence, targeting PHB1 and its tyrosine phosphorylation may be a novel therapeutic strategy for the treatment of diseases involving a dysregulation of PI3K signaling pathways.

## Phosphorylation of PHB1 and Akt signaling

Alpha serine/threonine protein kinase (Akt, also known as protein kinase B) is responsible for mediating various biological responses such as cell growth, cell differentiation, and apoptosis. It is a major signaling molecule present downstream of PI3 kinase.^40^ For the activation of Akt, phosphorylation at the Thr-308 residue is required; to attain its maximal activity, phosphorylation at Ser-473 is also required.^41^ Dysregulation of Akt activity is known to be involved in various tumor malignancies and other diseases.^42^ Interestingly, PHB1 has been identified as a target protein for Akt.^40^ In human pancreatic cancer cells, PHB1 has been shown to be phosphorylated by Akt.^40^ In these cells, Akt-induced phosphorylation of PHB1 occurs at Thr-258, which is located within the Akt consensus motif R-x-R-x-x-S⁄T in PHB1 protein. Although Thr-258 is not conserved in PHB2, it has been reported that Akt can phosphorylate Ser-91 and Ser-176, which are present in Akt consensus motifs.^43^ Phosphorylation of PHB1 by Akt can potentially impact various cellular functions of PHB1 and its stability. It is also possible that the Akt phosphorylation of PHB1 can impact its tyrosine phosphorylation at Tyr-114 and vice versa. We have shown that the phosphorylation of PHB1 at Thr-258 and Tyr-114 has opposing effects on insulin signaling.^22^ The phosphorylation of PHB1 at Thr-258 upregulates insulin signaling, and substitution of Thr-258 by isoleucine results in both the downregulation of Thr-308 and Ser-473 phosphorylation in Akt.^22^ Furthermore, the phosphorylation of PHB1 at Tyr-114 downregulates insulin signaling, and substitution of Tyr-114 by phenylalanine results in upregulation of Akt phosphorylation at Thr-308 and Ser-473.^22^ These results provided evidence that Akt phosphorylation of PHB1 impacts its phosphorylation at Tyr-114 and vice versa. Consistent with previous findings, a recent report has demonstrated that Akt phosphorylates PHB1 at Thr-258 in human bladder cancer cells.^44^ Phosphorylation of PHB1 by Akt promotes its mitochondrial localization and leads to the proliferation of bladder cancer cells.^44^ Furthermore, substitution of Thr-258 by alanine induced cell death in bladder cancer cells.^44^ These results demonstrate that PHB1 is an important regulator during bladder cell tumorigenesis and that the phosphorylation of PHB1 at Thre-258 plays a key role in this process. In another report, the levels of plasma membrane-associated PHB1 and migration-inducting gene-7 (*MIG-7*) were positively correlated with advanced stages of cancer in human lung tissues.^45^ These results suggest that Akt phosphorylates PHB1 at Thr-258, and phosphorylation of PHB1 plays an important role in the development and progression of different types of human lung cancers. Thus, PHB1 serves as a target protein for Akt and plays a role in regulating Akt activity. Consistent with this notion, downregulation of PHB1 phosphorylation and MIG-7 expression results in reduced cancer invasion and metastasis in human lung cancer xenograft mice.^45^ These results suggest that targeting the phosphorylation of PHB1 and MIG-7 can be effective in treating patients with advanced lung cancers.

Furthermore, PHB1 has been shown to physically interact with Raf and activate Ras-induced Raf/MAP/ERK signaling upon EGF stimulation.^29^ Raf-1 is normally phosphorylated at Ser-259 under inactive conditions, but upon EGF stimulation, Raf-1 is phosphorylated at Ser-338 and becomes activated. In this context, it is important to note that Thr-258 in PHB1 and Ser-259 in Raf-1 are located in the Akt consensus motif R-x-R-x-x-S⁄T.^46^ It is therefore conceivable that Thr-258 phosphorylation in PHB1 may compete with the Ser-259 phosphorylation of Raf-1 thereby reducing the phosphorylation of Raf-1 and facilitating its activation to promote cancer cell proliferation. In one recent report, it has been demonstrated that phosphorylation of PHB1 at Thr-258 and Tyr-259 correlated with the invasiveness and metastasis of human cervical cancer cells.^37^ A reduction in the invasiveness of cancer cells is observed when phosphorylation is absent on both of these residues. Interestingly, substitution of Tyr-259 for phenylalanine in PHB1 decreases the phosphorylation of Thr-258, but substitution of Thr-258 for isoleucine has no effect on the phosphorylation of Tyr-259.^37^ Thus, the phosphorylation at Tyr-259 in PHB1 appears to have a regulatory role in the phosphorylation at the Thr-258 site. It has been shown that a Thr-258 phospho-mutant form of PHB1 prevents Raf-1 activation,^37^ indicating that the phosphorylation at Thr-258 plays a crucial role in the induction of Raf-1 activation. Hence, activation of Raf-1/ERK signaling leads to an enhancement of the invasiveness and metastatic capabilities of cancer cells. It is anticipated that molecules targeting PHB1 and its phosphorylation on the plasma membrane may be effective in decreasing cancer cell metastasis and invasiveness. All of the above experimental findings highlight the crucial role played by PHB1 phosphorylation in cancer cells. However, more diligent studies are required to determine the role of PHB1 and its phosphorylation at serine/threonine residues by Akt as well as their relationship with tyrosine phosphorylation of PHB1.

## Tyrosine phosphorylation of PHB1 in T cell signaling

There are a number of reports that have identified PHB1 as a membrane-associated protein in T cells and have demonstrated its role in T-cell receptor signaling.^12,23,47^ Stimulation of murine T cells with anti-CD3 and anti-CD28 antibodies remarkably induces the cell surface expression of PHB1,^12^ which presumably participates in the T-cell receptor-mediated signaling cascade. PHB1 has been shown to be an endogenous ligand for sialic acid-binding lectin-9 (siglec-9) and negatively regulates T-cell signaling.^47^ Furthermore, *Salmonella typhi,* which causes systemic infection and typhoid in humans, targets PHB1 on T cells and downregulates T-cell-mediated immune signaling.^48,49^ In a separate study, to identify differentially expressed proteins upon activation of human T cells, Ross *et al.*^23^ found that the expression levels of PHB1 and PHB2 mRNA and protein were highly upregulated upon T-cell activation. Using proteomic analysis, the authors provided convincing evidence that PHB1 and PHB2 form a phosphocomplex in the inner mitochondrial membrane of primary human T cells.^23^ During this process, PHB1 is phosphorylated at serine residues, and PHB2 is phosphorylated at both serine and tyrosine residues.^23^ Furthermore, functional studies revealed that the PHB1/PHB2 phosphocomplex is required for the survival of differentiated T cells.^23^ It has been suggested that the phosphorylation of PHB2 at Tyr-248 may be important for protein-protein interactions because PHB2 Tyr-248 lies within a conserved NPXY motif, which could potentially interact with the phospho-tyrosine binding domain.^23^ Further analysis revealed that the PHB1/PHB2 phosphocomplex is required for mitochondrial homeostasis in T cells.^23^ It is possible that phosphorylation of PHB1/PHB2 at other tyrosine residues may also have a role in T-cell maturation, which warrants further investigations.

## Tyrosine phosphorylation of PHB1 in antigen-stimulated signaling in mast cells

Mast cells play a key role in the process of inflammation.^50^ They are the major effector cells that drive allergic responses. Upon stimulation with antigens, mast cells secrete a large number of granules that contain histamine and other inflammatory mediators such as cytokines and eicosanoids.^51,52^ In antigen-stimulated mast cell signaling, antigen binds to the IgE bound to FcεRI at the plasma membrane.^51,52^ The presentation of antigen to IgE results in the aggregation of FcεRI complexes, and the rapid tyrosine phosphorylation of immunoreceptor tyrosine-based activation motifs (ITAM) of the beta and the gamma chains of FcεRI by the Src family of tyrosine kinases, such as Lyn.^51,52^ Tyrosine phosphorylation of the gamma chain of FcεRI by Lyn leads to the recruitment of another cytoplasmic tyrosine kinase, Syk, to the gamma chain of FcεRI.^51,52^ The activation of Syk is essential for the activation of important signaling molecules such as transmembrane adaptor protein linker for the activation of T cells (LAT), phospholipase C (PLC), Src homology 2 (SH2) domain-containing leukocyte protein 76 (SLP-76) and Gab 2.^52^ These cascades of signaling events lead to the release of inflammatory markers that can induce allergic responses.

It has been shown that PHB1 is abundant in the intracellular granules of mast cells and has a critical role in antigen-stimulated allergic responses.^52^ Exposure of antigen to IgE results in translocation of PHB1 from the granules to the plasma membrane and subsequent tyrosine phosphorylation of PHB1 by Lyn.^52^ These experimental findings also suggest that PHB1 acts as a scaffold or adaptor protein to facilitate the phosphorylation of the FcεRI gamma complex by Lyn.^52^ Point mutation studies have revealed that phosphorylation at Tyr-114 and Tyr-259 is required for the recruitment of Syk to the FcεRI gamma complex and for the activation of mast cells.^52^ These experimental findings highlight that the phosphorylation of PHB1 at Tyr-114 and Tyr-259 plays a crucial role in the association of PHB1 with the membrane, where it acts as a scaffold protein for the FcεR gamma and Syk complex during antigen-stimulated signaling events in mast cells. Similar to our previous findings in 3T3-L1 and C2C12 cells,^18^ a Cys-69 mutant of PHB1 was unable to translocate to the plasma membrane and failed to participate in the antigen-stimulated mast cell signaling.^52^ Collectively, these studies suggest that the palmitoylation of PHB1 at Cys-69 is required for its translocation to the membrane and/or membrane association. More studies are required to understand the compartment-specific functions of PHB1 in different immune cell types.

## Phosphorylation of PHB1 and B-cell signaling

The association of PHB1 with the cell membrane of B-lymphocytes was first discovered over 20 years ago.^53^ PHB1 was shown to interact with the IgM receptor present on B-lymphocytes.^53^ However, the functional significance of PHB1-IgM association was not explored further until recently. It has now been shown that PHB1/PHB2 and the cytoplasmic domain of CD86 cooperate in mediating CD86 signaling in B-lymphocytes.^54^ CD86 is a transmembrane glycoprotein associated with B cells, dendritic cells and macrophages.^54,55^ In one study, Lucas *et al.*^54^ sought to identify potential signaling intermediates that associate with CD86, as well as their role in CD86 signaling pathway in B-lymphocytes. Under normal conditions, the expression level of CD86 was found to be low in resting B-cells. However, upon engagement with B-cell receptor CD-40 or IL-4 receptor, the levels of CD86 expression increase in B-cells.^56–59^ Unlike other transmembrane signaling molecules, CD86 contains a short cytoplasmic domain that is devoid of tyrosine phosphorylation motifs. By using a proteomic approach, the authors identified that in the CD40/IL-4-primed B cells, there is an interaction between PHB1, PHB2 and CD86.^54^ CD86 engagement on the surface of the primed B cells activates two signaling pathways, which leads to the activation of NF-κB.^59^ Inhibition of these signaling cascades in B cells decreases the expression of Oct-2, which in turn affects the rate of IgG1 production.^59^ Taken together, these results demonstrate that both PHB1/PHB2 and an intact CD86 cytoplasmic domain are required to mediate CD86 signaling, which regulates the level of IgG1 production by B cells. In these reports, however, the authors did not identify the residues that are phosphorylated in PHB1. Based on the available information in the literature as discussed above, it is possible that the phosphorylation of PHB1 at tyrosine residues (Tyr-114 and Tyr-259) may be critical in CD86 signaling in primed B cells. Future point mutation studies can provide clues regarding the role of tyrosine phosphorylation of PHB1 in primed B-cell signaling. In another report, Paris *et al.*^60^ have shown that Syk and PHB1 can interact with each other in a phosphorylation-dependent manner in antigen receptor-mediated B-cell signaling. It was found that PHB1 becomes phosphorylated at tyrosine residues and participate in immune signaling, but the specific phosphorylation site remains to be identified.^60^ As there are only four tyrosine residues in PHB1, it is likely that the phosphorylation occurs at previously known phosphorylation sites (Tyr-114, Tyr-249 and Tyr-259), which have been identified in a number of cell types and tissues including immune cells.^46,52^ In addition, whether the role of PHB1 in B-cell requires intracellular trafficking remains unclear. More studies are required to understand the compartment-specific functions of PHB1 in immune cell signaling in different immune cell types. Moreover, a role for PHB1 in TGF-β and STAT3 signaling pathways has also been reported.^30,31^ However, the potential involvement of tyrosine phosphorylation of PHB1 in these pathways has not yet been explored.

## FSH signaling pathway and the role of tyrosine phosphorylation of PHB1

Ovarian granulosa cells (GCs) play a vital role in the growth and development of the ovarian follicles.^13^ PHB1 is widely expressed in the ovary and its expression is dependent on the age of the organism and the stage in follicular development.^13^ In a recent study, it was shown that follicle-stimulating hormone (FSH) upregulates the expression of PHB1 in rat primary GCs.^13^ In addition, PHB1 was phosphorylated at Tyr-249, Thr-258 and Tyr-259 sites during GC differentiation.^13^ The observed level of phosphorylation at all three residues was found to be low in the absence of FSH stimulation, whereas enhanced phosphorylation was observed in the presence of FSH. Furthermore, phosphorylation of PHB1 was inhibited by the use of PD98059, which is an inhibitor of the MAPK-ERK pathway.^13^ These experimental results collectively suggest that PHB1 is a substrate of MEK1 and p38MAPK signaling during GC differentiation. It is interesting to note that Tyr-249 of PHB1 is located within the Raf-1 binding domain of PHB1, which spans through residues 243–275 in PHB1. Reports have demonstrated that PHB1 is indispensable for the activation of Raf-MAP-ERK pathway by Ras.^29^ A low level of Raf-1 Ser-338 phosphorylation and a high level of Ser-259 phosphorylation have been observed under low PHB1 conditions.^29^ Membrane targeting and activation of C-Raf by Ras require PHB1 *in vivo*.^29^ In the absence of PHB1, C-Raf kinase fails to interact with Ras upon activation by epidermal growth factor stimulation.^29^ These results indicate that there may be a hierarchical relationship between PHB1 and the MAPK-ERK pathway, and tyrosine phosphorylation of PHB1 might have an important role in regulating the Ras/Raf-mediated MAPK-ERK pathway. Cell fractionation studies have shown that only mitochondrial PHB1 is phosphorylated in rat granulosa cells,^13^ which would imply that the phosphorylation of PHB1 occurs in specific cellular compartments and/or has a role in the intracellular trafficking of PHB1. Taken together, these results provide new evidence that tyrosine phosphorylation of PHB1 plays an important role in regulating the FSH signaling pathway in ovarian granulosa cells.

## Role of Tyr-114 in iron binding by PHB1

**Figure 4:**
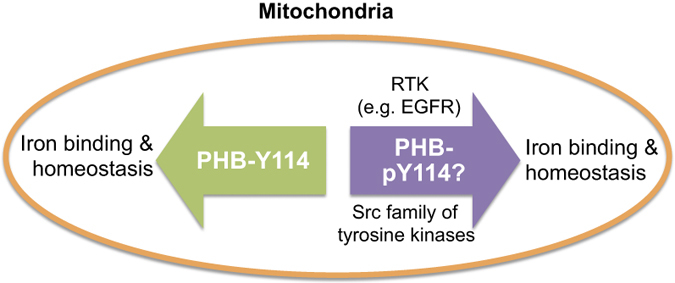
Does tyrosine phosphorylation of PHB1 plays a role in its iron binding function or potentially in other mitochondrial attributes of PHB1? A schematic diagram showing a potential role of the phosphorylation of PHB1 at tyrosine (for example, Y114) residue(s) in the iron binding function of PHB. This may be because a number of tyrosine kinases (for example, the RTK and Src family kinases) that phosphorylate PHB are known to localize to the mitochondria. RTK -receptor tyrosine kinase; p -phosphorylated; Y—tyrosine.

Mitochondria play a central role in iron homeostasis. They are unique sites for heme synthesis and major site for iron-sulfur cluster biosynthesis.^61^ The cellular proteins that are involved in iron homeostasis are often upregulated during oxidative stress. PHB1 has been reported to bind iron and be upregulated during oxidative stress. Specifically, Tyr-114 in PHB1 is an important residue for iron binding.^62^ There are numerous reports in the literature that provide support for PHB1 having a protective role in oxidative stress and mitochondrial dysfunction.^10,63,64^ It is possible that the iron-binding function of PHB1 plays a role in its protective effects against oxidative stress. We propose that the Tyr-114 residue in PHB1 has functions that are unique to each cellular compartment. For example, plasma membrane-associated PHB1 may be involved in cell signaling through tyrosine phosphorylation of PHB1, whereas the same tyrosine residue in mitochondrial PHB1 may aid in the iron binding function of PHB1. However, in light of new evidence showing the tyrosine phosphorylation of PHB1 in the mitochondrial fraction of granulosa cells,^13^ a possibility for the potential role of the phosphorylation of PHB1 at tyrosine residues in iron binding in mitochondria cannot be ruled out. This is because a number of tyrosine kinases, including EGFR, FGFR, ErB2, can translocate to the mitochondria, and the mitochondrial localization of Src family kinases has been reported.^65,66^ It is plausible that the phosphorylation status of Tyr-114 modulates the iron-binding property of PHB1 and thereby affects iron homeostasis in mitochondria or potentially other mitochondrial attributes of PHB such as chaperone/scaffolding functions (Figure 4). Moreover, we have recently shown that PHB1 has an important role in immune functions at the organismal level.^67^ However, the underlying mechanism involved remains to be determined. It is possible that mitochondria-specific functions of PHB1 have a role in immune functions.

## Mito-Ob and m-Mito-Ob mice: transgenic mouse models of PHB1

PHB1 has an important role in adipogenesis.^9^ We have shown that overexpression of PHB1 in 3T3-L1 pre-adipocytes induces adipocyte differentiation,^9^ whereas silencing of PHB1 in 3T3-L1 cells inhibits adipocyte differentiation.^68^ Micro-RNA-27, which targets PHB1, impairs adipocyte differentiation and mitochondrial function when it is expressed in adipose-derived stem cells.^69^ These results provide convincing evidence that PHB1 plays an important role in adipogenesis. In an attempt to find the role of PHB1 in adipose tissue biology at the systemic level, we developed a transgenic mouse (Mito-Ob) overexpressing PHB1 in adipocytes. As expected, Mito-Ob mice developed obesity independent of diet.^70^ Interestingly, female Mito-Ob mice had normal glucose homeostasis and insulin sensitivity, whereas male Mito-Ob mice developed impaired glucose homeostasis, hyperinsulinemia and insulin resistance.^70^ These results provide evidence that PHB1 has roles in regulating adipose tissue homeostasis and in sex-dimorphic functions.

As mentioned before, PHB1 is phosphorylated at Tyr-114 in response to insulin, and this modification aids in the recruitment of Shp1, which is a negative regulator of insulin signaling.^21^ Reports in the literature have also suggested that tyrosine phosphorylation of PHB1 has an important role in immune signaling, maturation and survival of T cells and in thymic growth.^23,71^ More recently, PHB1 has been identified as an adapter protein in antigen receptor signaling in mast cells.^52^ There, it facilitates protein-protein interactions in a Tyr-114 phosphorylation-dependent manner and modulates cytokine production. These results emphasize that phosphorylation of PHB1 at Tyr-114 is crucial for controlling cell signaling-mediated events. To explore the physiological relevance of phosphorylation of PHB1 in immune functions at the systemic level, we developed a mutant mouse (m-Mito-Ob) overexpressing Y114F-PHB1 from the *aP2* gene promotor.^28^ The *aP2* gene is primarily expressed in adipocytes, but is also expressed specifically in macrophages and dendritic cells.^67^ Similar to PHB1 mice, m-Mito-Ob mice developed obesity independent of diet in a sex-neutral manner, but only male mice developed glucose intolerance, hyperinsulinemia and insulin resistance, such as the Mito-Ob mice.^28^ These experimental results suggest that Tyr-114-mutant mice retain adipogenic functions and tyrosine phosphorylation may not have a role in mitochondria-associated adipogenic functions of PHB1. The Mito-Ob and m-Mito-Ob male mice exhibited similar metabolic features such as obesity, insulin resistance, and adipose tissue inflammation; however, male Mito-Ob mice developed non-alcoholic steatohepatitis (NASH) and hepatocellular carcinoma (HCC) by 12 months of age,^72^ whereas male m-Mito-Ob mice developed lymph node tumors by 6 months of age.^28^ Development of histiocytosis with massive lymphadenopathy in m-Mito-Ob mice demonstrated that Tyr-114 phosphorylation of PHB1 had anti-proliferative effects on immune cells such as macrophages and dendritic cells,^28^ and consequently on B and T cells. This is consistent with previous reports that showed Tyr-114 phosphorylation has an inhibitory effect on P13K/Akt signaling.^21,22^ Interestingly, ovariectomy in female m-Mito-Ob mice resulted in glucose intolerance, hyperinsulinemia and insulin resistance and they developed lymph node tumors similar to male m-Mito-Ob mice.^28^ These events were not observed in ovariectomized Mito-Ob mice.^28^ These results demonstrate that PHB1 and mutant-PHB1 lacking a Tyr-114 phosphorylation site act differently in the presence and absence of estrogen.^73^ It would be interesting to determine if the tyrosine phosphorylation of PHB1 has a role in estrogen-related cell signaling events and functions.

Although the mechanisms involved in PHB1- and mutant PHB1-induced metabolic and immune phenotypes in Mito-Ob and m-Mito-Ob mice remain to be elucidated, potential roles exist for the interaction of PHB1 with OGT and subsequent *O*-GlcNAc modification of PHB1 itself as well as insulin and immune signaling intermediates. This is because *O*-GlcNAc modifications of a number of insulin signaling intermediates have been shown to cause insulin resistance,^74–76^ and emerging evidence suggests that *O*-GlcNAc modification of immune signaling intermediates modify protein activity during cellular activation and differentiation.^77^ For instance, *O*-GlcNAc modification of the NF-κB subunit c-Rel is required for its DNA binding activity and subsequent cytokine expression in activated T cells.^78^ The inhibition of *O*-GlcNAc modification in this context is sufficient to repress IL-2 and IFNG expression following TCR stimulation. *O*-GlcNAc modification of STAT3 exerts an inhibitory effect on STAT3 phosphorylation, which results in a loss of IL-10 production in macrophages,^79^ thus demonstrating how *O*-GlcNAc modifications can control important transcriptional programs in immune cells. Of note, PHB1 has been shown to modulate NF-κB and STAT3 signaling.^80^ The focus has been on phosphorylation-mediated events. However, a potential role of *O*-GlcNAc modification and its crosstalk with phosphorylation has not yet been investigated.

## Adult-onset type 1 diabetes in male m-Mito-Ob

Obesity and its associated abnormalities appear to be an important factor in the development of adult-onset type 1 diabetes (T1D). However, the mechanisms behind the association of obesity and adult-onset T1D are not clearly understood. One surprising finding from Mito-Ob and m-Mito-Ob mouse models is that male m-Mito-Ob mice developed autoimmune insulitis on a high-fat diet but male Mito-Ob mice did not.^81^ Histological analysis of pancreatic islets from m-Mito-Ob mice revealed that there was a progressive increase in the infiltration of immune cells to the pancreatic islets. At early stage, immune cells were present around the islets in male m-Mito-Ob mice, indicating peri-insulitis.^81^ At advanced stages, immune cells migrated into the islets indicating features of intra-insulitis.^81^ Immunohistochemical studies revealed that immune cells present around the islets were positive for CD8 and F4/80,^81^ which indicated the presence of lymphocytes and macrophages around the pancreatic islets. However, pancreatic islets from the female m-Mito-Ob mice fed a high-fat diet and male m-Mito-Ob mice fed a low-fat diet were negative for CD8 and F4/80.^81^ These experimental findings indicate that PHB1 may play a role in immune checkpoints and that Tyr-114-mutant PHB1 may function as a genetic susceptibility factor in adult-onset T1D. More importantly, these findings provide evidence of both the crucial role played by phosphorylation of PHB1 at Tyr114 in the regulation of immune cell function and the role that a loss of tyrosine phosphorylation plays in the activation of diabetogenic effector T cells in response to environmental triggers or stress, such as a high-fat diet. Thus, Mito-Ob and m-Mito-Ob mice have created unique opportunities to gain new insights into the role of obesity-related abnormalities in the adult-onset T1D and for defining the role of PHB1 in antigen presenting cells such as dendritic cells and macrophages. Dendritic cells and macrophages are important cells in the immune system involved in antigen presentation and processing.^55,56^ These cells act as mediators between the adaptive and innate immune systems. In our transgenic models, both male and female Mito-Ob and m-Mito-Ob mice overexpress PHB1 and mutant PHB1, respectively, in dendritic cells and macrophages, but only male mice develop immune dysregulation suggesting a sexually dimorphic role of PHB1 in these cell types.^67,73^ These results also suggest an important role of PHB1 phosphorylation at Tyr-114 in regulating the function of dendritic cells and macrophages. It is possible that mutant PHB1 can affect macrophage and dendritic cell function in a sexually dimorphic manner or that mutant PHB1-overexpressing macrophages and dendritic cells respond differently in male and female organisms. In summary, these findings provide a proof-of-concept that that obesity-related abnormalities promote the development of adult-onset T1D by coupling environmental factors with genetic susceptibility. It is possible that similar factors are involved in the development of adult-onset T1D in humans. It is anticipated that the Mito-Ob and m-Mito-Ob mice will prove to be valuable tools for mechanistic studies uncovering the role of PHB1 in cell signaling pathways.

## Conclusions and future perspectives

PHB1 has pleiotropic functions, such as cell proliferation, cell growth, cell signaling and cell death. Most of these cellular events require proper signaling mechanisms to function effectively. This review emphasizes several of the recent developments in the study of PHB1 associated with cell signaling events and highlights the role of PHB1 tyrosine phosphorylation in mediating these cell-signaling processes (Figure 3). Defects in the tyrosine phosphorylation of PHB1 can lead to the development of various diseases, such as different types of cancer and diabetes.^28,70,72,81^ In this review, we have highlighted the regulatory role of tyrosine phosphorylation of PHB1 in the insulin signaling pathway, the MAPK/ERK pathway, the FSH signaling pathway, in immune cell signaling, and potentially in iron binding in mitochondria. Emerging evidence suggests that role of tyrosine phosphorylation of PHB1 in signaling mechanisms is complex. Moreover, several studies have shown that PHB1 upon stimulation with various stimuli, translocates to the plasma membrane and becomes phosphorylated^18,21,22^ and further traffics between the plasma membrane, mitochondria and the nucleus.^10,38,39^ It is possible that phosphorylation of PHB1 play a role in its intracellular trafficking. It is crucial to determine more regarding the cellular compartment-specific functions of PHB1, and the relationship between the PHB1 that is present on the plasma membrane, in the mitochondria and the nucleus. The two novel transgenic mouse models that we developed have revealed hidden roles of PHB1 and its tyrosine phosphorylation in adipocyte and immune crosstalk in immunometabolism. We anticipate that a fuller understanding of the roles and regulation of PHB1, especially the phosphorylation of its different tyrosine residues may lead to the discovery of new therapeutic targets including signal transduction-targeting therapy for metabolic- and immune-related diseases.
